# Dietary intake is independently associated with the maximal capacity for fat oxidation during exercise^1^,^2^

**DOI:** 10.3945/ajcn.116.133520

**Published:** 2017-03-01

**Authors:** Gareth Fletcher, Frank F Eves, Elisa I Glover, Scott L Robinson, Carlijn A Vernooij, Janice L Thompson, Gareth A Wallis

**Affiliations:** 3 School of Sport, Exercise and Rehabilitation Sciences, University of Birmingham, Birmingham, United Kingdom; and; 4 GlaxoSmithKline Consumer Healthcare, Brentford, United Kingdom

**Keywords:** health, metabolism, nutrition, physical activity, substrate oxidation

## Abstract

**Background:** Substantial interindividual variability exists in the maximal rate of fat oxidation (MFO) during exercise with potential implications for metabolic health. Although the diet can affect the metabolic response to exercise, the contribution of a self-selected diet to the interindividual variability in the MFO requires further clarification.

**Objective:** We sought to identify whether recent, self-selected dietary intake independently predicts the MFO in healthy men and women.

**Design:** The MFO and maximal oxygen uptake (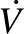
O_2_ max) were determined with the use of indirect calorimetry in 305 healthy volunteers [150 men and 155 women; mean ± SD age: 25 ± 6 y; body mass index (BMI; in kg/m^2^): 23 ± 2]. Dual-energy X-ray absorptiometry was used to assess body composition with the self-reported physical activity level (SRPAL) and dietary intake determined in the 4 d before exercise testing. To minimize potential confounding with typically observed sex-related differences (e.g., body composition), predictor variables were mean-centered by sex. In the analyses, hierarchical multiple linear regressions were used to quantify each variable’s influence on the MFO.

**Results:** The mean absolute MFO was 0.55 ± 0.19 g/min (range: 0.19–1.13 g/min). A total of 44.4% of the interindividual variability in the MFO was explained by the 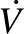
O_2_ max, sex, and SRPAL with dietary carbohydrate (carbohydrate; negative association with the MFO) and fat intake (positive association) associated with an additional 3.2% of the variance. When expressed relative to fat-free mass (FFM), the MFO was 10.8 ± 3.2 mg · kg FFM^−1^ · min^−1^ (range: 3.5–20.7 mg · kg FFM^−1^ · min^−1^) with 16.6% of the variability explained by the 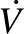
O_2_ max, sex, and SRPAL; dietary carbohydrate and fat intakes together explained an additional 2.6% of the variability. Biological sex was an independent determinant of the MFO with women showing a higher MFO [men: 10.3 ± 3.1 mg · kg FFM^−1^ · min^−1^ (3.5–19.9 mg · kg FFM^−1^ · min^−1^); women: 11.2 ± 3.3 mg · kg FFM^−1^ · min^−1^ (4.6–20.7 mg · kg FFM^−1^ · min^−1^); *P* < 0.05].

**Conclusion:** Considered alongside other robust determinants, dietary carbohydrate and fat intake make modest but independent contributions to the interindividual variability in the capacity to oxidize fat during exercise. This trial was registered at clinicaltrials.gov as NCT02070055.

## INTRODUCTION

The capacity to oxidize fat [fat oxidation (FAT-OX)^5^] as a fuel is important for metabolic health, weight management, and body composition. For instance, the skeletal muscle of patients with type 2 diabetes displays an impaired ability to oxidize fat (1). In addition, a high respiratory quotient, which is indicative of low FAT-OX relative to carbohydrate oxidation, is predictive of both future body mass gain (2–4) and the regain of fat mass (FM) after diet-induced reductions in body mass (5). Exercise acutely increases both energy expenditure and FAT-OX, and the capacity to oxidize fat during exercise is related to daily FAT-OX and insulin sensitivity (6). Therefore, a further understanding of the factors that influence FAT-OX during exercise could help to optimize the use of physical activity for the maintenance of metabolic health, body mass, and body composition (7).

The pattern of fuel utilization during exercise that has been performed under a variety of experimental conditions has been well characterized (8–12). However, substantial interindividual variability in energy substrate partitioning and the maximal rate of fat oxidation (MFO) during exercise has been identified (9, 12, 13). A previous study attributed 36% of the interindividual variability in the MFO to aerobic fitness [maximal oxygen uptake (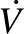
O_2_ max)], self-reported physical activity level (SRPAL), body composition [fat-free mass (FFM) and FM], and sex (9). Dietary intake could contribute to the observed interindividual variability in the MFO, although its relative influence has not been previously quantified to our knowledge.

A high-fat diet (particularly a ketogenic diet) can substantially increase FAT-OX during exercise, whereas an isoenergetic high-carbohydrate diet can reduce FAT-OX (12, 14, 15). However, the outcomes of studies that have explored divergent macronutrient intakes may not be applicable to the habitual dietary patterns that have been reported by the majority of the population (16, 17). Previously, a significant inverse relation between self-selected dietary fat intake and the exercising respiratory exchange ratio has been reported (18). This observation has provided important insight, but the relatively small sample size, use of an exercise-trained cohort, and limited range of exercise intensities studied precluded a full exploration of the influence of the diet on FAT-OX across a broad spectrum of active individuals.

An understanding of the independent contribution of a diet relative to other known contributors to FAT-OX represents an important step. A diet is a modifiable variable, which could enhance FAT-OX during exercise. Because the MFO occurs at moderate intensities, the diet could influence FAT-OX at exercise intensities that are consistent with current public health recommendations with broad implications for public health. Therefore, the primary aim of this study was to determine the extent to which the diet independently predicted the interindividual variability in the MFO during exercise in healthy young men and women. In addition, it has been reported that women exhibit greater rates of FAT-OX than do men during exercise (19), and yet the role of biological sex, independent of other factors that are relevant to substrate oxidation [e.g., 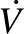
O_2_ max, SRPAL, body size–related variables (9)], on the MFO has not previously been assessed to our knowledge.

## METHODS

### Participants

Between January 2013 and March 2014, 377 individuals were assessed for their eligibility to participate in the study with 364 subjects (181 men and 183 women) meeting the inclusion criteria. Data collection was completed by March 2014. Participants were recruited from the surrounding local community via postal notices, e-mails, and word of mouth. Participants were excluded from taking part in the study for the following reasons: if they were <18 or >45 y old; had BMI (in kg/m^2^) <18.8 or >29.9; were >192 cm in height [i.e., maximum dual-energy X-ray absorptiometry (DXA) scanning height]; were taking any medication or supplements that had the potential to interfere with normal metabolism (e.g., β-blockers, insulin, bronchodilators, anti-inflammatory agents, and thyroxine); were completely sedentary; were current or recent (within 30 d) smokers; were engaged in prolonged periods of food abstinence; or were pregnant, breast feeding, or amenorrheic combined with not using hormonal contraception. Participants provided written informed consent in accordance with the Helsinki Declaration of 1975 as revised in 1983 to take part in the study that was approved by the National Research Ethics Service Committee East Midlands, Northampton, United Kingdom (reference 12EM0470). This trial was registered at clinicaltrials.gov as NCT02070055.

**FIGURE 1:**
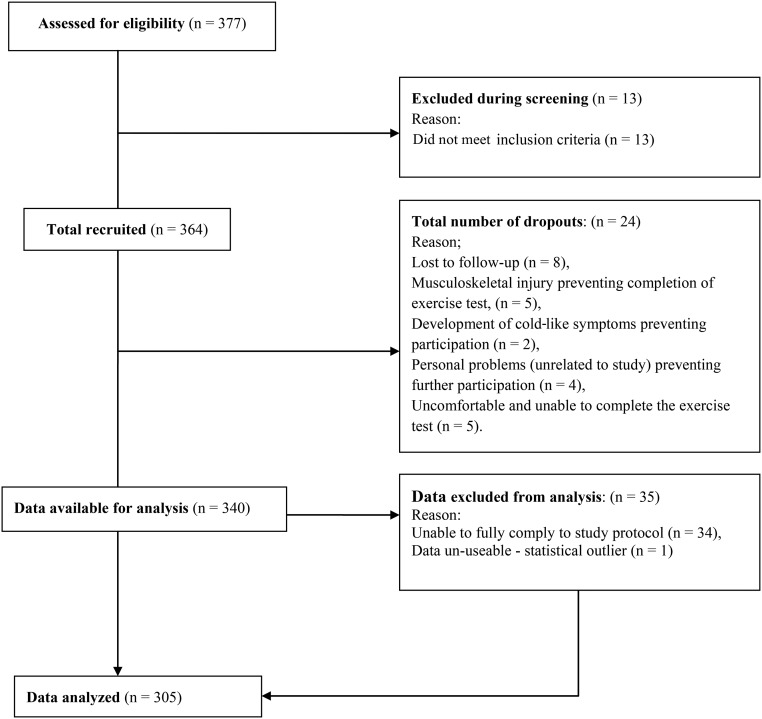
Flowchart of participant recruitment and involvement in the study.

A total of 305 (150 men and 155 women) participants completed the study, which met the a priori objective to achieve a similar number as in previous work (9). A flowchart of participant recruitment and involvement in the study is shown in **Figure 1**. Participants (*n* = 24) withdrew after providing consent for the following reasons: musculoskeletal injury preventing completion of exercise testing (*n* = 5), development of cold or flu-like symptoms (*n* = 2), personal reasons unrelated to study (*n* = 4), uncomfortable with the exercise testing (*n* = 5), and lost to follow-up (*n* = 8). Data from 34 subjects were excluded from the analysis because the subjects were unable to fully comply with the study protocol. Data from one subject were excluded because of statistical grounds (Statistical Analysis).

Characteristics of the 305 participants who completed the study are shown in **Table 1**. All volunteers were deemed to be healthy as was assessed with the use of a general health questionnaire. Women also completed a self-report questionnaire to estimate the menstrual cycle phase during which testing occurred and to document hormonal contraceptive (HC) use [follicular: *n* = 57 (HC use: *n* = 19); luteal: *n* = 82 (HC use: *n* = 30); and amenorrheic and taking HCs: *n* = 16].

**TABLE 1:** Participant demographic characteristics, ethnicity, aerobic capacity, and physical activity levels^1^

Variable	All subjects (*n* = 305)	Men (*n* = 150)^2^	Women (*n* = 155)
Age, y	25 ± 6^3^	24 ± 7 (18–45)^4^	25 ± 6 (18–45)
Height, m	1.72 ± 0.09	1.78 ± 0.06*	1.66 ± 0.06
Mass, kg	68.7 ± 11.1	76.0 ± 9.1***	61.6 ± 7.9
BMI, kg/m^2^	23.0 ± 2.0	23.9 ± 2.3***	22.2 ± 2.2
WC, cm	78 ± 8	82 ± 7***	73 ± 7
Body fat, %	24.7 ± 7.1	19.6 ± 4.8***	29.7 ± 5.1
FFM, kg	51.2 ± 10.8	60.1 ± 7.2***	42.6 ± 5.1
Fat mass, kg	16.5 ± 5.0	14.8 ± 4.6***	18.2 ± 4.8
Visceral adipose tissue, g	227 ± 123	274 ± 118***	181 ± 110
A:LB FM^5^	0.17 ± 0.06	0.20 ± 0.06***	0.14 ± 0.04
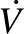 O_2_ max			
* *L/min	3.44 ± 0.83	4.11 ± 0.57***	2.80 ± 0.46
mL · kg · min^−1^	49.9 ± 8.0	54.4 ± 6.9***	45.6 ± 6.6
* *mL · kg FFM^−1^ · min^−1^	67.1 ± 7.0	68.5 ± 7.0***	65.7 ± 6.7
SRPAL	1.57 ± 0.13	1.58 ± 0.14	1.56 ± 0.12
Ethnicity, *n*			
White	230	109	121
Black	12	4	8
Asian, Indian, or Pakistani	27	18	9
Chinese or other Asian	23	8	15
Mixed	13	11	2

1 A:LB FM, ratio of abdominal to lower-body fat mass; FFM, fat-free mass; SRPAL, self-reported physical activity level; 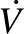
O_2_ max, maximal oxygen uptake; WC, waist circumference.

2 *^,^***Significantly different from women (determined with the use of an independent *t* test unless otherwise stated): *P < 0.05, ***P < 0.001.

3 Mean ± SD (all such values).

4 Mean ± SD; range in parentheses (all such values).

5 Determined with the use of a Mann-Whitney *U* test.

### Study design

The current study followed a single-center, cross-sectional design with each participant attending the laboratory on 2 occasions that were separated by 5–10 d. At the first visit, demographic data were recorded before undertaking a familiarization exercise test (identical to the one described in the Exercise test section later in Methods). Participants were provided with digital weighing scales and a food and physical activity diary that they completed during the 4 consecutive days before the second visit to the laboratory. Subjects were instructed to maintain their normal dietary habits and physical activity levels during this time with the exception of the day before the second visit for which they refrained from strenuous physical activity and alcohol consumption. After a 10–12 h overnight fast except for water intake, participants attended the laboratory for the second visit between 0700 and 0900 at which time anthropometric measurements and body composition were determined. Thereafter, participants completed an exercise test to determine the submaximal exercise substrate utilization, MFO, and 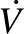
O_2_ max with the use of indirect calorimetry.

### Four-day dietary and physical activity assessment

Participants were provided with comprehensive written and verbal instructions that explained how to complete a 4-d weighed food diary. Digital weighing scales, one of which was portable and pocket sized (Digital Pocket Mini Gold; Swees) and one of which was standard sized (Digital LCD Electronic Kitchen Weighing Scales 10 kg; Macallen) were provided to allow all food and drink consumed to be weighed to the nearest gram. Each food diary was checked by the first author (GF) with any uncertainties clarified by the participant. Diaries were analyzed (by GF) with the use of Dietplan 6.70.67 software (Forestfield Software Ltd.) to produce a comprehensive report of energy and nutrient intakes. When a consumed food item was missing from the database, the nutritional data were located from the manufacturer and entered manually.

An adapted version of the physical activity record that was designed by Bouchard et al. (20) was used to estimate energy expenditure with the use of the factorial approach (21). Briefly, subjects were required to record their level of physical activity every 15 min with the use of a code that was provided from a 12-point scale, with each point on the scale having a designated physical activity level (SRPAL) value. A daily SRPAL value was determined from the total amount of time that was spent at each of the assigned codes per day. Total daily energy expenditure was estimated by multiplying the obtained daily SRPAL (22) value by the resting metabolic rate that was estimated with the use of the Harris-Benedict equation (23) with the energy balance calculated as energy expenditure minus energy intake (EI). The mean values from the 4-d measurement period were calculated for energy expenditure, energy balance, and dietary intake data.

### Anthropometric measures and body-composition assessment

After voiding and while wearing minimal clothing, participants were weighed to the nearest 10 g (Champ II scales; Ohaus) and height was measured to the nearest centimeter (Stadiometer; Seca). Waist circumference was measured to the nearest millimeter with the tape and measured midway between the uppermost border of the iliac crest and the lower border of the costal margin (rib cage). Body composition was determined with the use of DXA (Discovery QDR W series; Hologic) with a manufacturer-recommended phantom scan that was performed daily for calibration and quality-control assurance. In addition, the ratio of abdominal to lower-body fat mass (A:LB FM) was determined from the DXA scan in a similar manner as was previously described (24) but that differed in the use of an automated compared with manual determination of abdominal fat (APEX version 4.0; Hologic Inc.).

### Exercise test

Participants were familiarized with the exercise test during visit 1 to ensure that, during visit 2, the physiologic and metabolic responses that were measured were as near normal and maximal as possible and not overly influenced by the performance of a novel task. The motorized treadmill (PPS 70sport-I; Woodway/Quasar; h/p/cosmos)–based exercise test was adapted from that used previously by Achten et al. (25). The test commenced at a speed of 3.5 km/h and a gradient of 1% [to reflect the oxygen cost of outdoor running (26)], and the speed was increased by 1 km/h every 3 min until a respiratory-exchange ratio of 1.00 was reached and, therefore, FAT-OX was negligible thereafter (27). The treadmill speed was kept constant with the gradient increasing by 1%/min until volitional exhaustion to determine the 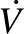
O_2_ max within the same protocol. Heart rate was recorded continuously via telemetry with the use of a heart-rate monitor (Polar S610i; Polar Electro Ltd.). Mean ± SD environmental conditions during testing were a relative humidity of 45% ± 7% and a temperature of 20°C ± 2°C. An electronic fan was positioned behind participants for use upon request.

A face mask (7450 V2; Hans Rudolph) was securely fitted, and breath-by-breath respiratory measurements [minute ventilation, oxygen uptake (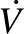
O_2_), and carbon-dioxide production] were recorded throughout the test with the use of an automated gas-analysis system (Oxycon Pro; CareFusion UK Ltd.). Gas analyzers were calibrated immediately before each exercise test according to the manufacturer’s recommendations with the use of calibration gases (5.07% CO_2_ and 14.79% O_2_) (BOC Gases), and the volume transducer was manually calibrated with a 3-L bidirectional syringe (Jaegar). The highest rolling 60-s mean 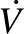
O_2_ measurement was considered to be maximal (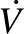
O_2_ max) if 2 of the 3 following conditions were met: *1*) a plateau (an increase of ≤2 mL · kg^–1^ · min^–1^) in 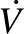
O_2_ with a further increasing workload; *2*) a heart rate ≤10 beats/min of the age-predicted maximum (for men: 220 beats/min minus age; for women: 206 beats/min minus 0.88 (age) (28)]; and *3*) a respiratory-exchange ratio >1.1. If a plateau in 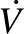
O_2_ did not occur, a 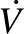
O_2_ peak value was obtained, which was defined as the highest mean 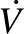
O_2_ measured over a 30-s period. The mean 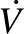
O_2_ and carbon-dioxide production during the final minute of each 3-min submaximal stage of the exercise test was used to calculate FAT-OX and carbohydrate oxidation with the use of the stoichiometric equations of Frayn (29) under the assumption of negligible urinary nitrogen losses. The highest attained rate of FAT-OX was identified as the MFO, and the exercise intensity (i.e., percentage of 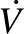
O_2_ max) associated with this rate was identified as the exercise intensity that elicited the maximal rate of fat oxidation (FatMax) (10). With the use of Matlab software (Matlab 2011a; MathWorks), carbohydrate and FAT-OX rates were determined for each 5% increment of 35–85% 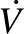
O_2_ max by interpolation (1000 points) between subsequent recorded data points and by logging the oxidation value nearest to the increment of 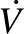
O_2_ max.

### Statistical analysis

Data were analyzed with the use of SPSS statistical package for Windows software (version 20.0; SPSS Inc.) and the R statistical software package (version 3.3.0; R Foundation for Statistical Computing). Data were checked for normality with the use of distribution plots and the Kolmogorov-Smirnov test. Differences in carbohydrate and FAT-OX rates between men and women across different exercise intensities were tested with a 2-factor repeated-measures ANOVA. Differences between men and women in baseline characteristics that were normally distributed were assessed with the use of independent sample *t* tests, and Mann-Whitney *U* tests were used for data that were nonnormally distributed. Significant sex-based differences were shown across most baseline characteristics (Tables 1 and **2**). Therefore, all independent variables were mean-centered by sex. This transformation allowed for tests to be conducted of the effects of sex independent of sex-related differences in baseline characteristics that were relevant to substrate oxidation (e.g., aerobic fitness, body-size related variables, and diet). Analyses used hierarchical multiple regression with previously identified variables [FM, FFM (for the absolute MFO only), sex, SRPAL, and 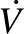
O_2_ max] (9) entered on step 1 and carbohydrate, fat, and protein intake (for the absolute MFO only) entered on step 2 to quantify the independent influence of each variable on the MFO expressed in absolute terms as g/min and relative to FFM, which was expressed as mg · kg FFM^−1^ · min^−1^. Variables were prescreened for multicollinearity; if a pair of variables had a Pearson’s *r* >0.85, one variable was eliminated on the basis of a priori expectations. Variables with a tolerance value <0.35 or a variance inflation factor >3 were also eliminated as was one influential case that was identified by having a Cook’s distance >1 (30). All results are expressed as means ± SDs unless otherwise stated with statistical significance accepted at *P* < 0.05.

**TABLE 2:** Macronutrient intake and related variables^1^

Variable	All subjects (*n* = 305)	Men (*n* = 150)	Women (*n* = 155)
Energy expenditure, kcal/d	2568 ± 445	2912 ± 348***	2236 ± 219
Energy balance, kcal/d	75 ± 553	103 ± 615	47 ± 486
Energy intake, kcal/d	2608 ± 738	3001 ± 719***	2227 ± 527
Contribution to energy intake, %			
Fat	34.3 ± 6.9	34.2 ± 7.0	34.4 ± 6.9
Protein	17.3 ± 5.8	19.1 ± 7.0***	15.5 ± 3.6
Carbohydrate	45.4 ± 9.3	43.1 ± 9.7***	47.5 ± 8.3
Alcohol^2^	2.6 ± 4.7	3.3 ± 4.9***	2.1 ± 4.3
Total, g/d			
Fat	99 ± 34	113 ± 33***	86 ± 28
Protein	113 ± 49	141 ± 52***	86 ± 26
Carbohydrate	313 ± 104	347 ± 114***	281 ± 82
Alcohol^2^	11 ± 21	15 ± 25***	7 ± 15

1 All values are means ± SDs. ***Significantly different from women, *P* < 0.001 (determined with the use of an independent *t* test unless otherwise stated).

2 Determined with the use of a Mann-Whitney *U* test.

## RESULTS

### Participant characteristics and nutritional intake

Participant characteristics and their nutritional intakes are reported in Tables 1 and 2, respectively. As expected from differences between the sexes in body size, men were significantly taller and heavier and had a larger waist circumference, greater BMI, lower-body fat (percentage), less FM, more FFM, a higher A:LB FM, and a greater 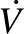
O_2_ max. Similarly, the total absolute EI and energy expenditure of men were higher than the values for women with men reporting greater absolute intakes of protein, fat, and carbohydrate as well as of alcohol. For men, there was a greater percentage contribution to total EI from protein and alcohol and less percentage contribution from carbohydrate than for women with no significant difference in fat intake. There were no differences between sexes in age, SRPAL, or energy balance.

### Substrate oxidation

**FIGURE 2:**
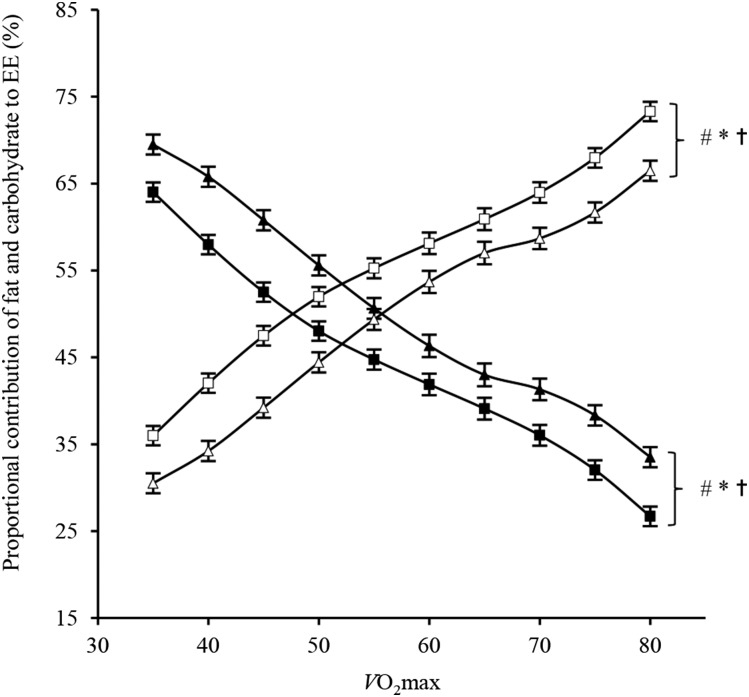
Sex differences in mean ± SEM proportional (percentage of total EE) contribution of rates of fat and carbohydrate oxidation during incremental exercise between 35% and 80% 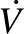
O_2_ max (men: *n* = 145; women: *n* = 135). The *x* axis shows 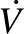
O_2_ max in percentages. Closed diamonds denote fat oxidation in women, and open diamonds denote carbohydrate oxidation in women; closed squares denote fat oxidation in men, and open squares denote carbohydrate oxidation in men. ^#^*P*-main effect of sex < 0.01, *P-main effect of exercise intensity < 0.001, and ^†^*P*-sex-by-intensity interaction = 0.049. *P* values were determined with the use of a 2-factor repeated-measures ANOVA. EE, energy expenditure; 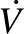
O_2_ max, maximal oxygen uptake.

The MFO was 0.55 ± 0.19 g/min (range: 0.19–1.13 g/min) or 10.8 ± 3.2 mg · kg FFM^−1^ · min^−1^ (range: 3.5–20.7 mg · kg FFM^−1^ · min^−1^) and occurred at 60% ± 16% (range: 19–92%) of the 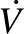
O_2_ max (FatMax). The absolute MFO was greater in men (0.62 ± 0.19 g/min; range: 0.21–1.13 g/min) than in women (0.48 ± 0.15 g/min; range: 0.19–0.99 g/min) (*P* < 0.0001), whereas the MFO was lower in men when expressed relative to FFM [men: 10.3 ± 3.1 mg · kg FFM^−1^ · min^−1^ (range: 3.5–19.9 mg · kg FFM^−1^ · min^−1^); women:, 11.2 ± 3.3 mg · kg FFM^−1^ · min^−1^ (range: 4.6–20.7 mg · kg FFM^−1^ · min^−1^); *P* < 0.05]. There was no statistically reliable difference (*P* = 0.09) in the exercise intensity at which the MFO occurred (i.e., FatMax) between men (58.7% ± 15.9%; range: 22.9–91.4%) and women (61.8% ± 15.7%; range: 19.3–92.3% of the 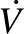
O_2_ max). The percentage contribution of FAT-OX and carbohydrate oxidation to energy expenditure for each sex is displayed in **Figure 2**. Proportionally, fat served as the main fuel source at low exercise intensities until ∼50% 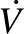
O_2_ max after which carbohydrate became the dominant source of energy. The proportional contribution of fat to energy expenditure was higher in women than in men with the opposite difference shown for carbohydrate (i.e., lower in women).

### Determinants of MFO

Analyses used a 2-step hierarchical regression with biological sex-, fitness-, and body-size–related variables that were expected to influence the MFO entered on step 1 and macronutrient intake variables entered on step 2. Macronutrient intake values were entered in absolute terms (grams per day) as opposed to the relative contribution to EI (percentage). When expressed as a percentage of EI, the macronutrients were inevitably linked to each other by the common divisor of the percentage of EI that was used in their derivation. If macronutrients were expressed as the percentage of EI, it would have been impossible to determine their independent contributions to the variability in MFO. The simple bivariate correlations between energy balance or the A:LB FM and MFO were not significant and, therefore, were not entered in the hierarchical regression (for MFO expressed as g/min or mg · kg FFM^−1^ · min^−1^: energy balance, *r* = 0.08, *P* = 0.18 and *r* = 0.09, *P* = 0.11, respectively; A:LB FM, *r* = −0.05, *P* = 0.35 and *r* = −0.08, *P* = 0.15, respectively).

The results of the hierarchical regression analysis with the dependent variable of the absolute MFO (grams per minute) are summarized in **Table 3**. 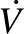
O_2_ max, SRPAL, and sex accounted for 44.4% of the variability in the MFO with no significant contribution from either FFM or FM. The step 2 analysis improved the amount of variability that was explained by 3.2% because of the significant and independent contributions of carbohydrate and fat intakes. The addition of the interaction terms for the macronutrients did not alter the independent contributions of carbohydrate and fat or further explain the variability in the MFO, and thus, these interactions are not included in Table 3 (*R*^2^ change = 0.01, *P* = 0.86). The relative magnitude of the standardized coefficients indicated the relative size and direction of their independent contribution. Positive standardized coefficients for 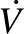
O_2_ max, SRPAL, and fat intake indicated that these variables were positive predictors of the MFO (i.e., an independent increase in any of these variables corresponded to a greater MFO). The negative standardized coefficient for carbohydrate intake indicated a reduction in the MFO after an increase in this variable with sex also having a negative association that reflected the dummy coding (men: one; women: 2).

**TABLE 3:** Hierarchical multiple linear regression for absolute MFO (*n* = 305)^1^

	Absolute MFO, g/min (*n* = 305)		
Step and independent variable	Standardized coefficient (95% CI)	*R*^2^ (bootstrapped 95% CI)	Adjusted *R*^2^
1		0.444 (0.368, 0.525)***^,2^	0.435
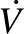 O_2_ max, L/min	0.477 (0.336, 0.617)***		
SRPAL	0.196 (0.096, 0.297)***		
Sex (M = 1; F = 2)	−0.385 (−0.470, −0.300)***		
FM, kg	−0.024 (−0.117, 0.069)		
FFM, kg	−0.091 (−0.222, 0.041)		
2		0.476 (0.409, 0.570)**^,2^	0.462
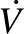 O_2_ max, L/min	0.527 (0.387, 0.667)***		
SRPAL	0.238 (0.135, 0.341)***		
Sex (M = 1; F = 2)	−0.385 (−0.468, −0.302)***		
FM, kg	−0.025 (−0.118, 0.067)		
FFM, kg	−0.115 (−0.248, 0.018)		
Protein intake, g	−0.046 (−0.145, 0.053)		
Carbohydrate intake, g	−0.178 (−0.276, −0.080)***		
Fat intake, g	0.144 (0.049, 0.239)**		

1 **^,^***Significance: **P < 0.01, ***P < 0.001. FFM, Fat-free mass; FM, fat mass; MFO, maximal rate of fat oxidation; SRPAL, self-reported physical activity level; 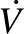
O_2_ max, maximal oxygen uptake.

2 Significant change in *R*^2^.

A similar 2-step hierarchical regression procedure was used for the MFO relative to FFM expressed as mg · kg FFM^−1^ · min^−1^ (**Table 4**). FFM could not be included in the analysis because of its contribution to the dependent variable. In addition, FM and protein intake were removed from the analysis because of their potentially spurious associations with the dependent variable (31). Both factors were independently associated with the divisor of the ratio, FFM per se (data not shown). Accordingly, in the final analysis, 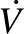
O_2_ max, SRPAL, and sex together accounted for 16.6% of the variability in the MFO. Carbohydrate intake made a significant (*P* = 0.01) independent contribution to the step 2 analysis with a trend (*P* = 0.062) for a similar role of fat intake as in the absolute MFO analysis, which together explained a further 2.6% of the variability. 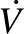
O_2_ max, SRPAL, sex (coding: men, 1; women, 2) and fat intake had positive relations with the MFO, whereas the association for carbohydrate intake was negative. The residual plots for analyses presented in Tables 3 and 4 are available online as **Supplemental Figures 1** and **2**, respectively.

**TABLE 4:** Hierarchical multiple linear regression for MFO relative to FFM^1^

	Relative MFO, mg · kg FFM^−1^ · min^−1^ (*n* = 305)		
Step and independent variable	Standardized coefficient (95% CI)	*R*^2^ (bootstrapped 95% CI)	Adjusted *R*^2^
1	—	0.166 (0.109, 0.271)***^,2^	0.158
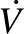 O_2_ max, L/min	0.131 (0.014, 0.249)*		
SRPAL	0.305 (0.187, 0.422)***		
Sex (M = 1; F = 2)	0.136 (0.033, 0.240)**		
2	—	0.192 (0.141, 0.301)**^,2^	0.178
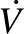 O_2_ max, L/min	0.155 (0.035, 0.276)**		
SRPAL	0.344 (0.221, 0.465)***		
Sex (M = 1; F = 2)	0.136 (0.034, 0.238)**		
Carbohydrate intake, g	−0.165 (−0.285, −0.046)**		
Fat intake, g	0.103 (−0.005, 0.211)^#^		

1 *^,^**^,^***^,#^Significance: *P < 0.05, **P < 0.01, ***P < 0.001, ^#^*P* = 0.062. FFM, Fat-free mass; MFO, maximal rate of fat oxidation; SRPAL, self-reported physical activity level; 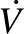
O_2_ max, maximal oxygen uptake.

2 Significant change in *R^2^*.

## DISCUSSION

The current study tested the influence of a recent, self-selected diet on the interindividual variability in the MFO during exercise in healthy young men and women. The total amount of variability explained (i.e., *R*^2^) was 47.6% and 19.2% of the absolute MFO expressed as g/min and the relative MFO expressed as mg · kg FFM^−1^ · min^−1^, respectively, with carbohydrate and fat intake accounting for ∼3% of this variation. The study also showed that biological sex was a determinant of the MFO independent of other important influences on FAT-OX. Although men had a higher absolute MFO (grams per minute) than did women, the reverse outcome was true when the MFO was expressed relative to FFM.

We confirmed that a substantial interindividual variability existed in the MFO, which ranged almost 6-fold (0.19–1.13 g/min; 3.5–20.7 mg · kg FFM^−1^ · min^−1^). Non–diet-related variables included in the analyses explained 44.4% and 16.6% of the variability in the absolute MFO expressed as g/min and the relative MFO expressed as mg · kg FFM^−1^ · min^−1^, respectively. These amounts were greater than those that were previously reported with the use of similar variables (36% and 13%, respectively) (9). In the current study, participants were fully familiarized with the testing procedures (compared with no familiarization), and the MFO was determined during exercise that was performed in the overnight-fasted state (compared with a minimum of 4 h postprandial). A detailed diary was used to assess physical activity rather than a brief questionnaire. Body composition was assessed with DXA scanning rather than according to the skin-fold technique. These methodologic improvements, which afforded a greater precision and reliability of measurements, accounted for the greater explained variance. In addition, the main objective of the study (i.e., dietary intake) explained a further ∼3% of the interindividual variability in the MFO during exercise.

Carbohydrate and fat intakes were the only dietary determinants of the MFO, which explained the variability in the MFO expressed in both absolute and relative terms (trend for dietary fat intake for relative MFO: *P* = 0.062). Higher carbohydrate intake was associated with a reduced MFO, which echoed controlled short-term dietary manipulation studies in which marked isoenergetic increases in dietary carbohydrate decreased FAT-OX (14, 32, 33). This result likely reflected a direct influence of carbohydrate intake on its subsequent availability for oxidation during exercise (32) or the related antilipolytic effect of insulin (34). An increase of glycolytic flux during exercise (e.g., with high exercise intensities or increased carbohydrate provision) can also directly downregulate mitochondrial long-chain FAT-OX (35, 36). The positive association of fat intake with the MFO was also consistent with previous studies in which high-fat or ketogenic diets substantially augmented FAT-OX or the MFO (12, 15, 18, 37–39). Fat intake has been suggested to influence FAT-OX through several mechanisms including greater plasma and intramuscular lipid availability (40, 41), greater expression of key proteins that are involved in cellular fatty acid uptake (37, 38, 42) and β oxidation (37, 43), and the reciprocal downregulation of enzymes (e.g., pyruvate dehydrogenase) that are involved in carbohydrate oxidation (44–46). Collectively, the current study clarified an independent role of both carbohydrate and fat intakes in the modulation of FAT-OX even within the context of typical dietary patterns.

Contrary to suggestions that a negative energy balance would increase FAT-OX (47), but consistent with the results of Rosenkilde et al. (48), we showed no relation between energy balance and the MFO. The potential limitations of the estimation of EI and EI expenditure with the use of a self-report in free-living individuals have been well documented (49, 50). Nonetheless, detailed activity diaries coupled with weighed food intakes should have provided relatively accurate data. In addition, the direction of relations for both carbohydrate and fat intakes with the MFO matched previous expectations. Also, we saw no impact of protein intake on the MFO in our cross-sectional cohort. One previous longitudinal study reported increases in the MFO from 0.43 ± 0.11 to 0.51 ± 0.11 g/min after 3 mo of protein supplementation, which did not occur in a control group (51). However, this finding should be interpreted with caution because a debatable statistical method supported the conclusion (i.e., within-group but not between-group changes were assessed) (52). Alternatively, the discrepancy with the current study in the suggested influence of protein could have reflected methodologic differences in designs (i.e., cross-sectional compared with longitudinal). Overall, our data suggest that a short-term (i.e., 4-d) energy balance and protein intake, when considered alongside other known determinants, are not substantial contributors to the interindividual variability in the MFO.

The variability in the MFO that was attributed to dietary carbohydrate and fat could appear to have been modest (∼3%), and thus the importance of this observation warrants consideration. Clearly, the large effects of aerobic fitness and the physical activity level on the MFO provided evidence of their importance when targeting FAT-OX for metabolic health. Nonetheless, we studied free-living participants under minimal dietary constraints. The variation between individuals occurred under conditions that mimicked their daily lives. The independent association of dietary intake on the MFO emerged in addition to factors that were previously shown to exert large effects. Thompson et al. (53) suggested that a ∼5% increase in FAT-OX while physically active could make important contributions to the maintenance of the daily fat balance. This suggestion was supported by our recent linking of the capacity for FAT-OX during exercise with 24-h FAT-OX (6). The findings presented in the current study, in a large, diverse, healthy population, suggest a dietary macronutrient manipulation could exert modest effects on FAT-OX during physical activity that are applicable to real-world settings.

A further key strength of our analysis is the minimization of the influence of typically observed sex-related differences in the measured independent variables. This minimization allowed us to make conclusions that were not previously possible (9) and to state with greater certainty that 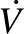
O_2_ max, SRPAL, and biological sex (i.e., women compared with men) are positive determinants of the MFO (9). Our data that showed a greater (∼10%) contribution of FAT-OX to exercise energy expenditure in women across a range of exercise intensities (Figure 2) were confirmatory (9, 19, 54), but the independent effect of biological sex on the MFO has not, to our knowledge, been previously reported after appropriate statistical adjustment for baseline sex-based differences. Note that higher FAT-OX was observed in women although men exhibited a small but significantly higher aerobic capacity (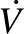
O_2_ max expressed as mL · kg FFM^−1^ · min^−1^) (Table 1). These results further highlight the independent role of sex-related differences as determinants of FAT-OX during exercise. Other physiologic differences that were unaccounted for in this study, such as ovarian hormones or intramuscular substrate availability, are possible candidates for enhanced FAT-OX in women (19, 55). Unfortunately, although menstrual cycle phases were self-reported by women participants, ovarian hormonal concentrations were not measured to confirm the menstrual phases, and as such, it was not appropriate to feature this variable in the current analysis. Finally, in contrast to previous reports, FFM, FM, or the locations of FM (i.e., the A:LB FM) were not independently associated with the MFO (9, 24). Our observations highlight the importance of both an adequate sample size and controlling for sex-based differences that may otherwise artificially influence conclusions that involve mix-sexed cohorts.

In conclusion, self-selected dietary intakes of fat and carbohydrate exert modest but independent influences on the maximal rate of FAT-OX during exercise. Biological sex is also an independent determinant with women having a higher MFO relative to FFM than that of men. Collectively, the study highlights the importance of modifiable lifestyle factors such as fitness, physical activity, and diet in determining FAT-OX during physical activity at intensities that are consistent with current public health recommendations with implications for the optimization of metabolic health, body mass, and body composition.

## Footnotes

5 Abbreviations used: A:LB FM, ratio of abdominal to lower-body fat mass; DXA, dual-energy X-ray absorptiometry; EI, energy intake; FatMax, exercise intensity eliciting the maximal rate of fat oxidation; FAT-OX, fat oxidation; FFM, fat-free mass; FM, fat mass; HC, hormonal contraceptive; MFO, maximal rate of fat oxidation; SRPAL, self-reported physical activity level; O_2_, oxygen uptake; O_2_ max, maximal oxygen uptake.

## References

[1] Kelley DE, Simoneau JA Impaired free fatty acid utilization by skeletal muscle in non-insulin-dependent diabetes mellitus. J Clin Invest 1994;94:2349–56. 7989591 10.1172/JCI117600 PMC330064

[2] Zurlo F, Lillioja S, Espositodelpuente A, Nyomba BL, Raz I, Saad MF, Swinburn BA, Knowler WC, Bogardus C, Ravussin E Low ratio of fat to carbohydrate oxidation as predictor of weight-gain: study of 24-H RQ. Am J Physiol 1990;259:E650–7. 2240203 10.1152/ajpendo.1990.259.5.E650

[3] Marra M, Scalfi L, Contaldo F, Pasanisi F Fasting respiratory quotient as a predictor of long-term weight changes in non-obese women. Ann Nutr Metab 2004;48:189–92. 15249759 10.1159/000079556

[4] Shook RP, Hand GA, Paluch AE, Wang X, Moran R, Hebert JR, Jakicic JM, Blair SN High respiratory quotient is associated with increases in body weight and fat mass in young adults. Eur J Clin Nutr 2016;70:1197–202. 26603877 10.1038/ejcn.2015.198

[5] Ellis AC, Hyatt TC, Hunter GR, Gower BA Respiratory quotient predicts fat mass gain in premenopausal women. Obesity (Silver Spring) 2010;18:2255–9. 20448540 10.1038/oby.2010.96 PMC3075532

[6] Robinson SL, Hattersley J, Frost GS, Chambers ES, Wallis GA Maximal fat oxidation during exercise is positively associated with 24-hour fat oxidation and insulin sensitivity in young, healthy men. J Appl Physiol (1985) 2015;118:1415–22. 25814634 10.1152/japplphysiol.00058.2015

[7] Brooks GA, Butte NF, Rand WM, Flatt JP, Caballero B Chronicle of the Institute of Medicine physical activity recommendation: how a physical activity recommendation came to be among dietary recommendations. Am J Clin Nutr 2004;79:921S–30S. 15113740 10.1093/ajcn/79.5.921S

[8] Brooks GA, Mercier J Balance of carbohydrate and lipid utilization during exercise: the “crossover” concept. J Appl Physiol (1985) 1994;76:2253–61. 7928844 10.1152/jappl.1994.76.6.2253

[9] Venables MC, Achten J, Jeukendrup AE Determinants of fat oxidation during exercise in healthy men and women: a cross-sectional study. J Appl Physiol (1985) 2005;98:160–7. 15333616 10.1152/japplphysiol.00662.2003

[10] Achten J, Gleeson M, Jeukendrup AE Determination of the exercise intensity that elicits maximal fat oxidation. Med Sci Sports Exerc 2002;34:92–7. 11782653 10.1097/00005768-200201000-00015

[11] Romijn JA, Coyle EF, Sidossis LS, Gastaldelli A, Horowitz JF, Endert E, Wolfe RR Regulation of endogenous fat and carbohydrate metabolism in relation to exercise intensity and duration. Am J Physiol 1993;265:E380–91. 8214047 10.1152/ajpendo.1993.265.3.E380

[12] Volek JS, Freidenreich DJ, Saenz C, Kunces LJ, Creighton BC, Bartley JM, Davitt PM, Munoz CX, Anderson JM, Maresh CM, Metabolic characteristics of keto-adapted ultra-endurance runners. Metabolism 2016;65:100–10. 26892521 10.1016/j.metabol.2015.10.028

[13] Achten J, Jeukendrup AE Maximal fat oxidation during exercise in trained men. Int J Sports Med 2003;24:603–8. 14598198 10.1055/s-2003-43265

[14] Helge JW, Wulff B, Kiens B Impact of a fat-rich diet on endurance in man: role of the dietary period. Med Sci Sports Exerc 1998;30:456–61. 9526894 10.1097/00005768-199803000-00018

[15] Phinney SD, Bistrian BR, Evans WJ, Gervino E, Blackburn GL The human metabolic response to chronic ketosis without caloric restriction: preservation of submaximal exercise capability with reduced carbohydrate oxidation. Metabolism 1983;32:769–76. 6865776 10.1016/0026-0495(83)90106-3

[16] Statistics NCHS, National Center for Health Statistics. Health, United States, 2015: with special feature on racial and ethnic health disparities. Washington (DC): US Government Printing Office; 2016. 27308685

[17] Bates B, Lennox A, Prentice A, Bates C, Page P, Nicholson S, Swan G. National Diet and Nutrition Survey results from years 1, 2, 3 and 4 (combined) of the rolling programme (2008/2009-2011/2012): a survey carried out on behalf of Public Health England and the Food Standards Agency. London; 2014.

[18] Goedecke JH, Gibson AS, Grobler L, Collins M, Noakes TD, Lambert EV Determinants of the variability in respiratory exchange ratio at rest and during exercise in trained athletes. Am J Physiol Endocrinol Metab 2000;279:E1325–34. 11093921 10.1152/ajpendo.2000.279.6.E1325

[19] Tarnopolsky LJ, MacDougall JD, Atkinson SA, Tarnopolsky MA, Sutton JR Gender differences in substrate for endurance exercise. J Appl Physiol (1985) 1990;68:302–8. 2179207 10.1152/jappl.1990.68.1.302

[20] Bouchard C, Tremblay A, Leblanc C, Lortie G, Savard R, Theriault G A method to assess energy expenditure in children and adults. Am J Clin Nutr 1983;37:461–7. 6829488 10.1093/ajcn/37.3.461

[21] Manore M, Meyer NL, Thompson J Sport nutrition for health and performance. 2nd ed. Champaign (IL): Human Kinetics; 2009.

[22] NRC (US). Recommended dietary allowances/Subcommittee on the tenth edition of the RDAs, Food and Nutrition Board, Commission on Life Sciences, National Research Council. Washington (DC): The National Academies Press; 1989.

[23] Harris JA, Benedict FG A biometric study of human basal metabolism. Proc Natl Acad Sci USA 1918;4:370–3. 16576330 10.1073/pnas.4.12.370 PMC1091498

[24] Isacco L, Duche P, Thivel D, Meddahi-Pelle A, Lemoine-Morel S, Duclos M, Boisseau N Fat mass localization alters fuel oxidation during exercise in normal weight women. Med Sci Sports Exerc 2013;45:1887–96. 23531714 10.1249/MSS.0b013e3182935fe3

[25] Achten J, Venables MC, Jeukendrup AE Fat oxidation rates are higher during running compared with cycling over a wide range of intensities. Metabolism 2003;52:747–52. 12800102 10.1016/s0026-0495(03)00068-4

[26] Jones AM, Doust JHA 1% treadmill grade most accurately reflects the energetic cost of outdoor running. J Sports Sci 1996;14:321–7. 8887211 10.1080/02640419608727717

[27] Jeukendrup AE, Wallis GA Measurement of substrate oxidation during exercise by means of gas exchange measurements. Int J Sports Med 2005;26 Suppl 1:S28–37. 15702454 10.1055/s-2004-830512

[28] Gulati M, Shaw LJ, Thisted RA, Black HR, Bairey Merz CN, Arnsdorf MF Heart rate response to exercise stress testing in asymptomatic women: the St. James women take heart project. Circulation 2010;122:130–7. 20585008 10.1161/CIRCULATIONAHA.110.939249

[29] Frayn KN Calculation of substrate oxidation rates in vivo from gaseous exchange. J Appl Physiol 1983;55:628–34. 6618956 10.1152/jappl.1983.55.2.628

[30] Field A Discovering statistics using IBM SPSS statistics. London: SAGE Publications Ltd; 2013.

[31] Kronmal RA Spurious correlation and the fallacy of the ratio standard revisited. J R Stat Soc Ser A Stat Soc 1993;156:379–92.

[32] Bergström J, Hermansen L, Hultman E, Saltin B Diet, muscle glycogen and physical performance. Acta Physiol Scand 1967;71:140–50. 5584523 10.1111/j.1748-1716.1967.tb03720.x

[33] Lambert EV, Speechly DP, Dennis SC, Noakes TD Enhanced endurance in trained cyclists during moderate intensity exercise following 2 weeks adaptation to a high fat diet. Eur J Appl Physiol Occup Physiol 1994;69:287–93. 7851362 10.1007/BF00392032

[34] Horowitz JF, Mora-Rodriguez R, Byerley LO, Coyle EF Lipolytic suppression following carbohydrate ingestion limits fat oxidation during exercise. Am J Physiol 1997;273:E768–75. 9357807 10.1152/ajpendo.1997.273.4.E768

[35] Coyle EF, Jeukendrup AE, Wagenmakers AJ, Saris WH Fatty acid oxidation is directly regulated by carbohydrate metabolism during exercise. Am J Physiol 1997;273:E268–75. 9277379 10.1152/ajpendo.1997.273.2.E268

[36] Sidossis LS, Gastaldelli A, Klein S, Wolfe RR Regulation of plasma fatty acid oxidation during low- and high-intensity exercise. Am J Physiol 1997;272:E1065–70. 9227453 10.1152/ajpendo.1997.272.6.E1065

[37] Cameron-Smith D, Burke LM, Angus DJ, Tunstall RJ, Cox GR, Bonen A, Hawley JA, Hargreaves M A short-term, high-fat diet up-regulates lipid metabolism and gene expression in human skeletal muscle. Am J Clin Nutr 2003;77:313–8. 12540388 10.1093/ajcn/77.2.313

[38] Helge JW, Watt PW, Richter EA, Rennie MJ, Kiens B Fat utilization during exercise: adaptation to a fat-rich diet increases utilization of plasma fatty acids and very low density lipoprotein-triacylglycerol in humans. J Physiol 2001;537:1009–20. 11744773 10.1111/j.1469-7793.2001.01009.x PMC2279002

[39] Webster CC, Noakes TD, Chacko SK, Swart J, Kohn TA, Smith JA Gluconeogenesis during endurance exercise in cyclists habituated to a long‐term low carbohydrate high fat diet. J Physiol 2016;594:4389–405. 26918583 10.1113/JP271934 PMC4967730

[40] Zderic TW, Davidson CJ, Schenk S, Byerley LO, Coyle EF High-fat diet elevates resting intramuscular triglyceride concentration and whole body lipolysis during exercise. Am J Physiol Endocrinol Metab 2004;286:E217–25. 14559721 10.1152/ajpendo.00159.2003

[41] Johnson NA, Stannard SR, Mehalski K, Trenell MI, Sachinwalla T, Thompson CH, Thompson MW Intramyocellular triacylglycerol in prolonged cycling with high- and low-carbohydrate availability. J Appl Physiol 2003;94:1365–72. 12626469 10.1152/japplphysiol.00833.2002

[42] Goedecke JH, Christie C, Wilson G, Dennis SC, Noakes TD, Hopkins WG, Lambert EV Metabolic adaptations to a high-fat diet in endurance cyclists. Metabolism 1999;48:1509–17. 10599981 10.1016/s0026-0495(99)90238-x

[43] Helge JW, Kiens B Muscle enzyme activity in humans: role of substrate availability and training. Am J Physiol 1997;272:R1620–4. 9176356 10.1152/ajpregu.1997.272.5.R1620

[44] Stellingwerff T, Spriet LL, Watt MJ, Kimber NE, Hargreaves M, Hawley JA, Burke LM Decreased PDH activation and glycogenolysis during exercise following fat adaptation with carbohydrate restoration. Am J Physiol Endocrinol Metab 2006;290:E380–8. 16188909 10.1152/ajpendo.00268.2005

[45] Peters SJ Regulation of PDH activity and isoform expression: diet and exercise. Biochem Soc Trans 2003;31:1274–80. 14641042 10.1042/bst0311274

[46] Putman CT, Spriet LL, Hultman E, Lindinger MI, Lands LC, McKelvie RS, Cederblad G, Jones NL, Heigenhauser GJ Pyruvate dehydrogenase activity and acetyl group accumulation during exercise after different diets. Am J Physiol 1993;265:E752–60. 8238502 10.1152/ajpendo.1993.265.5.E752

[47] Braun B, Brooks GA Critical importance of controlling energy status to understand the effects of “exercise” on metabolism. Exerc Sport Sci Rev 2008;36:2–4. 18156946 10.1097/jes.0b013e31815e42c2

[48] Rosenkilde M, Nordby P, Nielsen LB, Stallknecht BM, Helge JW Fat oxidation at rest predicts peak fat oxidation during exercise and metabolic phenotype in overweight men. Int J Obes (Lond) 2010;34:871–7. 20157319 10.1038/ijo.2010.11

[49] Schoeller DA Limitations in the assessment of dietary energy intake by self-report. Metabolism 1995;44(2 Suppl 2):18–22. 10.1016/0026-0495(95)90204-x 7869932

[50] Irwin ML, Ainsworth BE, Conway JM Estimation of energy expenditure from physical activity measures: determinants of accuracy. Obes Res 2001;9:517–25. 11557832 10.1038/oby.2001.68

[51] Soenen S, Plasqui G, Smeets AJ, Westerterp-Plantenga MS Protein intake induced an increase in exercise stimulated fat oxidation during stable body weight. Physiol Behav 2010;101:770–4. 20826169 10.1016/j.physbeh.2010.08.019

[52] Allison DB, Antoine LH, George BJ Incorrect statistical method in parallel-groups RCT led to unsubstantiated conclusions. Lipids Health Dis 2016;15:77. 27083538 10.1186/s12944-016-0242-3 PMC4869669

[53] Thompson D, Karpe F, Lafontan M, Frayn K Physical activity and exercise in the regulation of human adipose tissue physiology. Physiol Rev 2012;92:157–91. 22298655 10.1152/physrev.00012.2011

[54] Chenevière X, Borrani F, Sangsue D, Gojanovic B, Malatesta D Gender differences in whole-body fat oxidation kinetics during exercise. Appl Physiol Nutr Metab 2011;36:88–95. 21326382 10.1139/H10-086

[55] Lundsgaard AM, Kiens B Gender differences in skeletal muscle substrate metabolism - molecular mechanisms and insulin sensitivity. Front Endocrinol (Lausanne) 2014;5:195. 25431568 10.3389/fendo.2014.00195 PMC4230199

